# Allergen immunotherapy for allergic asthma: a systematic overview of systematic reviews

**DOI:** 10.1186/s13601-017-0160-0

**Published:** 2017-08-02

**Authors:** Felix Asamoah, Artemisia Kakourou, Sangeeta Dhami, Susanne Lau, Ioana Agache, Antonella Muraro, Graham Roberts, Cezmi Akdis, Matteo Bonini, Ozlem Cavkaytar, Breda Flood, Kenji Izuhara, Marek Jutel, Ömer Kalayci, Oliver Pfaar, Aziz Sheikh

**Affiliations:** 1 Centre for Environmental and Preventive Medicine, Wolfson Institute of Preventive Medicine, London, UK; 2 Barts and the London School of Medicine and Dentistry, Queen Mary University of London, London, UK; 3 Neonatal Unit, Homerton University Hospital NHS Foundation Trust, London, UK; 4 Department of Hygiene and Epidemiology, University of Ioannina School of Medicine, Ioannina, Greece; 5 Evidence-Based Health Care Ltd, Edinburgh, UK; 6 Charite Medical University, Berlin, Germany; 7 Department of Allergy and Clinical Immunology, Faculty of Medicine, Transylvania University Brasov, Brasov, Romania; 8 Food Allergy Referral Centre Veneto Region, University Hospital of Padua, Padua, Italy; 9 The David Hide Asthma and Allergy Research Centre, St Mary’s Hospital, Newport, Isle of Wight, UK; 10 NIHR Biomedical Research Centre, University Hospital Southampton NHS Foundation Trust and Faculty of Medicine, University of Southampton , Southampton, UK; 11 Faculty of Medicine, University of Southampton, Southampton, UK; 12 Swiss Institute for Allergy and Asthma Research, Davos, Switzerland; 13 Sapienza University Rome, Rome, Italy; 14 Department of Allergy and Clinical Immunology, Sami Ulus Maternity and Children Training and Research Hospital, Ankara, Turkey; 15 European Federation of Allergy and Airways Diseases Patients Association, Brussels, Belgium; 16 Saga Medical School, Saga, Japan; 17 Wroclaw Medical University, Wrocław, Poland; 18 Hacettepe University, Ankara, Turkey; 19 Department of Otorhinolaryngology, Head and Neck Surgery, Universitätsmedizin Mannheim, Medical Faculty Mannheim, Heidelberg University, Mannheim, Germany; 20 Center for Rhinology and Allergology, Wiesbaden, Germany; 21 Asthma UK Centre for Applied Research, Usher Institute of Population Health Sciences and Informatics, The University of Edinburgh, Edinburgh, UK

## Abstract

**Background:**

There is clinical uncertainty about the effectiveness and safety of allergen immunotherapy (AIT) for the treatment of allergic asthma.

**Objectives:**

To undertake a systematic overview of the effectiveness, cost-effectiveness and safety of AIT for the treatment of allergic asthma.

**Methods:**

We searched nine electronic databases from inception to October 31, 2015. Systematic reviews were independently screened by two reviewers against pre-defined eligibility criteria and critically appraised using the Critical Appraisal Skills Programme quality assessment tool for systematic reviews. Data were descriptively and thematically synthesized.

**Results:**

We identified nine eligible systematic reviews; these focused on delivery of AIT through the following routes: subcutaneous (SCIT; n = 3); sublingual (SLIT; n = 4); and both SCIT and SLIT (n = 2). This evidence found that AIT delivered by SCIT and SLIT can improve medication and symptom scores and measures of bronchial hyper-reactivity. The impact on measures of lung function or asthma control was however less clear. We found no systematic review level evidence on the cost-effectiveness of SCIT or SLIT. SLIT had a favorable safety profile when compared to SCIT, particularly in relation to the risk of systemic reactions.

**Conclusions:**

AIT has the potential to achieve reductions in symptom and medication scores, but there is no clear or consistent evidence that measures of lung function can be improved. Bearing in mind the limitations of synthesizing evidence from systematic reviews and the fact that these reviews include mainly dated studies, a systematic review of current primary studies is now needed to update this evidence base, estimate the effectiveness of AIT on asthma outcomes and to investigate the relative effectiveness, cost-effectiveness and safety of SCIT and SLIT.

## Introduction

Asthma is a major public health problem affecting over 300 million people worldwide [1]. Its prevalence and impact are particularly on the rise in urbanized regions. With a projected surge in the world’s urban population it is estimated that by 2025 an additional 100 million people may develop asthma [2]. Asthma is therefore set to become one of the world’s most prevalent chronic diseases.

Patho-physiologically, asthma is a chronic inflammatory disorder of the airways leading to airflow limitation and remodelling [3]. The resulting signs and symptoms are dyspnea, cough, chest discomfort and wheezing. Based on clinical and laboratory findings, different asthma phenotypes have been described [4]. This review focuses on allergic asthma. Allergic asthma is one of the best described asthma phenotypes. Allergic sensitization is a strong risk factor for asthma inception and severity in children and in adults [5]. Currently, there is no cure for asthma, but symptomatic control can be achieved in the majority of patients through a combination of short-acting bronchodilators and inhaled corticosteroids with minimal, if any, side-effects. Long-acting beta-2 agonists, anti-leukotrienes, anticholinergics, theophylline, anti-IgE antibodies and other biologic agents can be added to achieve asthma control in more severe cases [6].

Allergen immunotherapy (AIT) is the only class of treatment for respiratory allergy that has the potential to change the course of the disease. Its immunological mechanisms of action involve induction of allergen-specific immune tolerance. AIT for allergic asthma is therefore a potential therapeutic option in appropriately selected patients with allergic asthma.

The European Academy of Allergy and Clinical Immunology (EAACI) is in the process of developing the *EAACI Guidelines on Allergen Immunotherapy for Allergic Asthma*. Guideline recommendations will be informed by formal evidence syntheses of the literature. This article is an overarching synthesis of the systematic review evidence on the effectiveness, cost-effectiveness and safety of AIT in the management of allergic asthma. It will be followed by a review of the primary studies.

## Methods

A detailed description of our methods is available in the published systematic review protocol [7]. We therefore confine ourselves here to a summary of our methods.

### Search strategy

Electronic literature searches were conducted to retrieve systematic reviews that have been conducted in relation to AIT for allergic asthma from the following electronic databases: Medline, Embase, Cochrane Library, HTA, EED, CINAHL, ISI Web of Science, TRIP, Current controlled trials and Australian and New Zealand Clinical Trials registry.

A highly sensitive search strategy was developed, and validated study design filters were applied to retrieve articles pertaining to the use of AIT for allergic asthma from electronic bibliographic databases (“Appendix”). We used the systematic review filter developed at McMaster University Health Information Research Unit (HIRU) (http://hiru.mcmaster.ca/hiru/HIRU_Hedges_MEDLINE_Strategies.aspx#Reviews). Based on further abstract and full paper screening, all systematic reviews were identified and screened for inclusion. The searches were for articles published from inception of the databases up to 31st October 2015. No language restrictions were applied. All titles were uploaded into the systematic review software Distiller SR (Evidence Partners, Ottawa, Canada).

### Eligibility criteria

We were interested in systematic reviews of randomized controlled trials (RCTs) in which AIT for different allergens (e.g. pollens, mites, animal dander and cockroach) were administered through the subcutaneous (SCIT) or sublingual (SLIT) routes compared with placebo or any active comparator.

Participants of interest were patients of any age with a physician confirmed diagnosis of allergic asthma, plus evidence of clinically relevant allergic sensitization as assessed by an objective biomarker (e.g. skin prick test or specific-IgE), in combination with a history of asthma symptoms due to allergen exposure. Reviews that investigated participants with both asthma and rhinitis/rhinoconjuctivitis, but presented separate outcomes for the two conditions were also included.

The primary outcome of interest was the effectiveness—both short-term and long-term, where long-term was defined as persistence of benefit after discontinuation of treatment—of AIT as assessed by symptoms and/or medication scores.

Secondary outcomes of interest included asthma control, asthma specific quality of life, exacerbations, lung function, environmental exposure chamber or bronchial allergen challenge, cost-effectiveness and safety as assessed by local and systemic reactions.

### Selection procedures

Title and abstract screening was conducted independently by two reviewers (SD and FS) and for those that appeared to meet the inclusion criteria full-texts were independently retrieved and screened (AK and FA). Any disagreements were resolved through discussion or, if necessary, arbitration by a third reviewer (SD).

### Data extraction

Data were extracted independently in Distiller SR by two reviewers (FA and AK) using pre-defined criteria. Disagreements were resolved by discussion between the reviewers and where agreement could not be reached by arbitration with a third reviewer (SD).

### Quality assessment

Independent quality assessment of all systematic reviews was undertaken by two reviewers (FA and AK) using the Critical Appraisal Skills Programme (CASP) tool for systematic reviews [8]. Disagreements were resolved through discussion; if agreement could not be reached a third reviewer (SD) arbitrated.

### Synthesis of evidence

Abstracted data were included into descriptive tables that included information on search strategy, population characteristics, study design, quality assessment, exposure, outcomes and subgroup analyses. Because of multiple outcomes of this overview and since there is no consensus whether and how meta-analysis should be performed from systematic reviews, we did not undertake meta-analysis.

## Results

### Characteristics of included systematic review

**Fig. 1:**
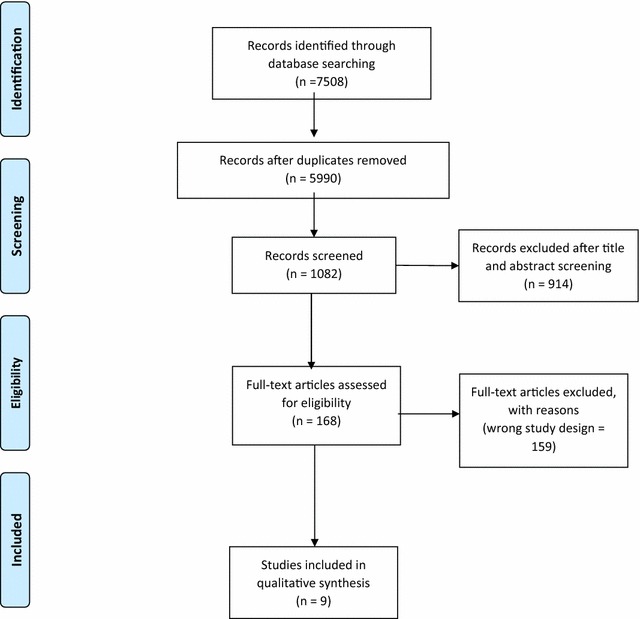
PRISMA diagram

Our searches retrieved nine systematic reviews, five of which included meta-analyses (see Fig. 1). The key features of these systematic reviews are summarized in Table 1.

**Table 1:** Main characteristics of included studies

Author, Year (Country)	Databases searched	Search period	No of studies relevant to this SR (No of participants)	Population	Intervention relevant to this SR	Allrgens	Outcomes relevant to this SR	Subgroup analyses relevant to this SR
Abramson, 2010 (Australia)	CENTRAL, MEDLINE, EMBASE, CINAHL, AMED, PsycINFO, and handsearching of journals and meeting abstracts	1950–2005	88 RCTs (3459)	adults and children with allergic asthma	SCIT vs placebo SCIT vs inhaled steroid	HDM, pollens, animal dander, moulds, chemically modified allergoids, antigen–antibody complexes	Effectiveness, lung function, non-specific BHR, allergen-specific BHR, safety	NR
Calamita, 2006 (Brazil)	MEDLINE, EMBASE,LILACS and the Cochrane Library	1966–2005	25 RCTS (1706)	adults and children with allergic asthma	SLIT vs placebo	Mites, pollens, mould, dander, latex	Effectiveness, lung function, non-specific BHR, safety	Age, treatment duration (no details)
Chelladurai, 2013 (USA)	MEDLINE, Embase, LILACS and the Cochrane Central Register of Controlled Trials	Up to December 21, 2012	4 RCTs (NR)	adults and children with allergic asthma	SCIT vs SLIT	Mites, pollens	Effectiveness, safety	NR
Compalati, 2009 (Italy)	MEDLINE, EMBASE, LILACS, SCOPUS	Up to March 31, 2008	9 DBRCTs (452)	adults and children with allergic asthma	SLIT vs placebo	HDM	Effectiveness	Age (children vs adults)
Erekosima, 2014 (USA)	MEDLINE, Embase, LILACS, and the Cochrane Central Register of Controlled Trials	1947-May 21, 2012	38 RCTs (NR)	adults and children with allergic asthma ± rhinitis/rhino conjunctivitis	SCIT vs any comparator	Mites, pollen, dander, mould	Effectiveness, lung function, allergen-specific BHR, quality of life, safety	NR
Kim, 2013 (USA)	Medline, Embase, LILACS, CENTRAL, and the Cochrane Central Register of Controlled Trials	Up to May 2012	27 RCTs (NR)	Children with allergic asthma ± rhinoconjuctivitis	SCIT vs any comparator SLIT vs any comparator SCIT vs SLIT	Dust mite, Rye, *Cladosporium, Alternaria*, Tree mix	Effectiveness, quality of life, safety	NR
Normansell, 2015 (UK)	CENTRAL, MEDLINE, EMBASE, CINAHL, AMED, PsycINFO, Clinical Trials.gov, WHO and handsearching of journals and meeting abstracts	Up to March 25, 2015	52 RCTs (5256)	adults and children with allergic asthma	SLIT vs placebo SLIT vs conventional pharmacotherapy	HDM, grass pollen, birch pollen, cockroach, cat dander, *Alternaria, Parietaria,* olive pollen, *Artemisia*, HDM + *Parietaria* (combination)	Effectiveness, Exacerbations, Quality of life, safety, BHR	Age (children vs adults)
Polzehl, 2006 (Germany)	MEDLINE and EMBASE	1970–2001	13 DBRCTs (442)	adults and children with allergic asthma	SCIT vs. placebo	HDM (D. pteronyssinus, D. farinae)	Effectiveness, safety, lung function, bronchial provocation	NR
Tao, 2013 (China)	PubMed, EMBASE and the Cochrane Central Register of Controlled Trials	Up to March 2012	16 DBRCTs (794)	adults and children with allergic asthma	SLIT vs placebo	Mite, pollen	Effectiveness, lung function, safety	Age (children vs adults)

The nine systematic reviews included 272 individual RCTs studying over 13,000 patients with asthma. Eight of the systematic reviews studied both children and adults [9–16]; whilst one focused on children only [17]. Two systematic reviews evaluated AIT for a single-allergen (i.e. house dust mite) [13, 17]. The remaining seven studied AIT for multiple allergens, these including animal dander, mold natural, pollens, modified allergens and latex [10–12, 14–16, 18]. Three systematic reviews evaluated SCIT [10, 14, 17], four evaluated SLIT [11, 13, 15, 16], and two examined a combination of SCIT and SLIT [12, 18].

The majority of the systematic reviews had primary outcomes which focused on asthma symptoms, medication usage, allergen-specific bronchial hyper-reactivity (BHR) and exacerbations with secondary outcomes of safety and disease specific quality of life (Table 1).

### Quality assessment

Two reviews by Abramson et al. and Normansell et al. were judged to be at low risk of bias [10, 15]. The remaining reviews were classified as being at moderate risk of bias (Table 2) [11–14, 16, 17].

**Table 2:** Quality assessment of included studies

Author, year	Focused question	Inclusion of appropriate studies	Inclusion of eligible studies	Quality assessment of studies	Appropriateness of synthesis	Overall results of review	Accuracy of results	Applicability to local populations	Considering all relevant outcomes	Benefits vs harms/costs	Overall risk of bias
Abramson	✓	✓	✓	✓	✓	✓	✓	n/a	✓	✓	Low
Calamita	✓	✓	Unclear	✓	✓	✓	✓	n/a	✓	Unclear	Unclear
Chelladurai	✓	✓	X	✓	✓	✓	✓	n/a	✓	✓	Unclear
Compalati	✓	✓	X	✓	✓	✓	✓	n/a	✓	✓	Unclear
Erekosima	✓	✓	X	✓	n/a	✓	✓	n/a	✓	✓	Unclear
Kim	✓	✓	X	✓	✓	✓	✓	n/a	✓	✓	Unclear
Normansell	✓	✓	✓	✓	✓	✓	✓	n/a	✓	✓	Low
Polzehl	✓	✓	✓	Unclear	✓	✓	✓	n/a	✓	✓	Unclear
Tao	✓	✓	X	✓	✓	✓	✓	n/a	✓	✓	Unclear

## SCIT focused reviews

### Asthma symptom scores

The strongest evidence for asthma symptom reduction was provided by the meta-analysis by Abramson et al., which included 88 RCTs of moderate quality randomizing a total of 3459 asthma patients [10]. Meta-analysis demonstrated a significant improvement in asthma symptoms scores based on data from thirty-five trials: the estimated standardised mean difference (SMD) for all allergens combined was −0.59 (95% CI −0.83 to −0.35) for AIT versus placebo, but there was high heterogeneity between studies (I^2^ = 73%). The authors concluded that it would have been necessary to treat three patients (NNT = 3; 95% CI 3–5) to avoid one person’s asthma symptoms deteriorating. Significant improvement of symptoms was most likely with AIT to pollen (NNT = 3; 95% CI 2–16); animal dander (NNT = 3; 95% CI 2–18) and other allergens such as molds, chemically modified allergoids or antigen–antibody complexes (NNT = 3; 95% CI 3–4). A smaller improvement was observed following HDM immunotherapy where six patients would need to be treated to avoid one deteriorating (NNT = 6; 95% CI 4–16). Only one trial directly compared AIT to inhaled corticosteroids and this found that symptoms improved more rapidly on inhaled steroids than AIT [18].

A further three reviews undertook a qualitative synthesis of the impact of AIT on asthma symptom scores. Erekosima et al. [14] reported on 10 studies [19–28], including 628 participants, that evaluated SCIT for control of asthma symptoms (eight compared SCIT to placebo, one compared SCIT to pharmacotherapy, and one compared SCIT to no SCIT); 90% of these studies reported a greater improvement in the SCIT arm than in the comparator arm. All of these trials used a single allergen: six were for HDM, one *Cladosporitum*, one timothy grass, one ragweed and one cat allergens. The review by Polzehl et al., which focused on the efficacy of SCIT with HDM extracts (*Dermatophagides pteronyssinus or farina)* in 442 adolescents and adults, reported that seven out of 12 studies showed a significant improvement in asthma symptom scores when compared to placebo [17].

Kim et al., using a narrative synthesis, reported evidence to demonstrate that SCIT improved asthma symptoms compared with placebo or pharmacotherapy [18]. This was only true however when using a single allergen; when multiple allergens were used, this was not the case. This evidence was from moderate to high quality studies.

### Asthma medication scores

The review by Abramson et al. pooled evidence from 21 studies to show that AIT significantly decreased medication usage with an SMD for all allergens combined versus placebo of −0.53 (95% CI −0.80 to −0.27) with moderate between study heterogeneity (I^2^ = 67%) [10]. Overall, it would have been necessary to treat four patients (NNT = 4; 95% CI 4–7) with AIT to avoid one patient requiring an increase in medication.

In Erekosima et al. [14], eight studies [18–22, 25, 29, 30], all using single allergen AIT, including 592 patients, reported on medication scores [18]. Five out of eight studies demonstrated a greater reduction in medication use in the SCIT group than in the comparator arm and two of the studies did not report the direction of change. Polzehl et al. [17] reported on asthma medication use in 10 studies: five of these showed a significant decrease in medication requirement after therapy whereas the remaining five studies showed no significant effect. Interestingly, no improvement was seen in four trials, which included patients with moderate-to-severe asthma.

Kim et al. presented results from four studies using single allergens which demonstrated that the SCIT group had a reduction in medication score greater than the control group [18]. A further study however reported the same scores for the treatment and placebo group.

### Asthma control and exacerbations

No results were available for these outcomes.

### Disease specific quality of life

Kim et al. reported quality of life outcomes in two SCIT studies [18]. Of these, a study of 50 patients with moderate risk of bias showed a significant improvement in quality of life in both patients and their parents and the other, a study of 300 patients with high risk of bias, showed no difference in SCIT and control groups.

### Lung function

In Abramson et al., of the 88 included studies 20 provided results for lung function [10]. Data for peak expiratory flow (PEF) and forced expiratory volume in one second (FEV1) were meta-analyzed. The overall results were inconclusive regarding the impact of AIT on lung function when compared to placebo: SMD for PEF 0.14 (95% CI −0.33 to 0.61) and SMD for FEV1 −0.32 (95% CI −0.96 to 0.31).with high between study heterogeneity (I^2^ = 81% for PEF; and I^2^ = 61% for FEV1) In seven studies reporting on lung function deterioration (simply as improved, worsened or the same), there was an overall trend implying lung function improvement after immunotherapy, but this did not reach statistical significance (RR 0.89; 95% CI 0.73–1.10).

In Erekosima et al., 11 studies enrolling 873 participants reported that the impact on lung function was ‘variable and inconsistent across studies’ (no further details provided) [14]. Polzehl et al., from nine studies, found no significant changes in lung function between SCIT and placebo [17].

### Environmental exposure chamber or bronchial allergen challenge

Three systematic reviews reported on bronchial allergen challenge . Meta-analysis results of 18 studies from Abramson et al. [10] showed that AIT reduced non-specific BHR following challenges when compared to placebo: overall SMD of −0.35 (95% CI −0.59 to −0.11). These effects were significant for methacholine: SMD of −0.25 (95% CI −0.51 to 0.00) and acetylcholine: SMD −1.29 (95% CI −2.28 to −0.31), but not for histamine: SMD −0.55 (95% CI −1.37 to 0.28) or cold air: SMD −0.52 (95% CI −1.31 to 0.26). Non-specific BHR was reported as increased, reduced or unchanged in five studies with an estimated RR for increased non-specific BHR of 0.48 (95% CI 0.33–0.72) favoring AIT.

In Erekosima et al., 13 studies reported on non-specific bronchial provocation tests on 568 participants in total [14]. Five out of 13 studies demonstrated greater improvement in the AIT group than the comparator. In Polzehl et al., two out of three studies showed significant improvement to methacholine challenge after 12–18 months [17].

Meta-analysis of 19 trials in the systematic review by Abramson et al. showed a significant reduction in allergen specific BHR following immunotherapy compared to placebo: mite SMD −0.98 (95% CI −1.39 to −0.58), pollen SMD −0.55 (95% CI −0.84 to −0.27), animal dander SMD −0.61 (95% CI −0.95 to −0.27), other allergens SMD −0.18 (95% CI −0.70 to 0.33) and overall SMD −0.61 (95% CI −0.79 to −0.43) [10]. Allergen specific BHR was reported as increased, reduced or unchanged in 16 studies with an estimated RR for increased allergen specific BHR of 0.51 (95% CI 0.41–0.63) favoring AIT.

In Erekosima et al., eight out of 11 studies demonstrated statistically significant improvement in the SCIT group that the comparator [14]. Finally, in Polzehl et al., three out of three studies showed significant improvement in the immunotherapy group [17].

### Safety

Abramson and colleagues in their systematic review reported on both local and systemic adverse reactions [10]. The pooled relative risk for local adverse reactions, as it was reported in ten studies, was 1.4 (95% CI 0.97–2.02) and homogenous (I^2^ = 0.0%). So, if sixteen patients were treated with immunotherapy, one would be expected to develop a local reaction. Systemic adverse reactions defined as anaphylaxis, asthma, rhinitis, urticaria or any combination of these were reported by 32 studies. The pooled relative risk for systemic reactions of any severity was 2.45 (95% CI 1.91–3.13) and relatively homogenous (I^2^ = 27%). They concluded that if nine patients were treated with immunotherapy, one would be expected to develop a systemic reaction.

Erekosima et al. reported data on safety from 35 trials [14]. Local reactions were reported in 11 studies, these showing that 71/346 (20.5%) patients in the SCIT arm experienced local reactions compared to 1/7 (14.3%) in the comparator arm. General reactions were reported in 14 studies, according to which 190/624 (30.4%) in the SCIT arm experienced general reactions, compared to 52/217 (24.0%) in the comparator arm. Finally anaphylactic reactions were reported in four studies and all events were concerning patients on immunotherapy (13/205 (6.3%) patients).The other study that reported on this outcome was Polzehl et al., which found that seven out of the thirteen studies showed no severe adverse events [17]. Four studies however, reported systemic adverse effects.

Kim et al. reported from 10 SCIT studies local reactions occurring at the injection site in both the treatment and placebo groups [18]. In terms of systemic reactions, bronchospasm occurred in 1–30% of patients and general systemic reactions in 3–34% of patients.

## SLIT focused reviews

### Asthma symptom scores

Evidence from the meta-analysis by Calamita et al. [11] which included 25 RCTs (19 double-blind and six open) showed a non-significant reduction in asthma symptoms in the SLIT arm compared to placebo: the SMD from meta-analysis of nine studies (enrolling 303 patients) t was −0.38 (95% CI −0.79 to 0.03).

The systematic review by Compalati et al., which included nine double-blind placebo controlled RCTs, found a significant reduction in symptom scores: SMD −0.95 (95% CI −1.74 to −0.15) compared to the placebo group; there was high heterogeneity (I^2^ = 92%) [13].

Normansell et al. found symptom scores in 42 studies, but only 17 presented these in a numerical fashion [15]. Of these studies, five showed no statistical significant difference between groups, nine studies reported statistically significant reductions in the SLIT group compared with the placebo group; two studies showed a small improvement and one study reported a marked reduction in symptoms during cat exposure for the SLIT group.

Tao et al. [16] which included 16 double-blind placebo controlled RCTs demonstrated that there was a significant reduction in patients symptom scores in the SLIT group compared to the placebo group: pooled SMD of −0.74 (95% CI −1.26 to −0.22), with significant between study heterogeneity (I^2^ = 91%). Subgroup analysis according to age showed that SMD was −0.87 (95% CI −1.54 to −0.21) in children and −0.40 (95% CI −1.36 to −0.25) in adults. Subgroup analysis by allergen showed a beneficial effect in the context of mite immunotherapy: SMD −0.97 (95% CI −1.69 to −0.25), but not pollen immunotherapy: SMD −0.29 (95% CI −0.96 to 0.38). Finally, subgroup analysis according to treatment duration showed that SMD was −0.96 (95% CI −1.69 to −0.22) for treatment less than 12 months whereas SMD was −0.60 (95% CI −1.30 to 0.10) for greater than 12 months of treatment.

Kim et al. using a narrative synthesis indicated that there was evidence to demonstrate that SLIT improved asthma symptoms compared with placebo or pharmacotherapy [18].

### Asthma medications scores

Meta-analysis results of six studies (with 254 patients) from Calamita et al. showed that there was a tendency towards improved medication scores favored by SLIT, but this was not conclusively shown: SMD of −0.91 (95% CI −1.94 to 0.12) with significant between studies heterogeneity (I^2^ = 91.8%) [11]. The results from Compalati et al., from seven studies enrolling 220 patients looking at dust mite allergen, however presented a significant (P = 0.02) reduction in rescue medication use: SMD −1.48 (95% CI −2.70 to −0.26) but significant heterogeneity (I^2^ = 96%).Subgroup analysis according to age, showed a significant reduction in children (SMD −1.86; 95% CI −3.34 to −0.38) but not in adults (SMD 0.23; 95% CI −0.33 to 0.78) [13]. Normansell et al. reported this outcome numerically in twelve studies, five of which reported favorably for the SLIT group and seven of which showed no statistically significant difference between groups [15]. Results from Tao et al., indicated that medication score was significantly (P = 0.02) reduced in the SLIT group compared to their comparators (SMD of −0.78; 95% CI −1.45 to −0.11), but there was significant heterogeneity (I^2^ = 93%) [16]. They also looked at children and adults separately and found that whereas in children there was a statistically significant reduction in medication score with SLIT (SMD −1.1; 95% CI −2.06 to −0.14, P = 0.03) this was not the case for adults (SMD −0.00; 95% CI −0.36 to 0.36; P = 0.99). They also indicated that prolonged duration of treatment did not have any additive beneficial effect: SMD for less than 12 month treatment was −0.98 (95% CI −2.14 to 0.19) and SMD for more than 12 months of treatment was −0.51 (95% CI −1.17 to 0.16).

Kim et al. included nine studies which reported on this outcome in relation to asthma: seven for HDM showed a significant improvement compared to placebo [18].

### Asthma control

Calamita et al. found that seven studies had reported on general asthma control, combining asthmatic symptoms, need for symptom relief medication, respiratory function test and lung hyper-reactivity, and found a significant improvement for AIT over placebo: RD −0.27 (95% CI −0.33 to −0.21) and RR 0.48 (95% CI 0.4–0.57). There was little heterogeneity between these studies (I^2^ = 36.3%) [11].

Tao et al. reported that two studies looked at ‘global improvement’, considering symptom remission, medication use and lung function, but they failed to identify any significant improvement (RR 3.31; 95% CI 0.25–44.44) [16].

Kim et al. reported two studies which looked at general asthma control [18]. One study found that after six months of SLIT to HDM compared to placebo, classification of asthma in the treatment group changed from ‘mild-moderate persistent to mild intermittent’. The second study however concluded that after three years of SLIT treatment there was no difference in the number of children with mild intermittent asthma when compared to the placebo group.

### Exacerbations

Normansell et al. reported on this outcome with data from one small study, enrolling 43 patients, which was assessed to be at high risk of bias [15]. It reported no exacerbations requiring emergency department attendance or hospital admission during the (four week) treatment period or the follow up period (5–6 weeks) in either the SLIT or placebo arms.

### Disease specific quality of life

Normansell et al. reported data on this outcome from five of the included studies, but no meta-analysis could be performed [15]. Results from these five studies were variable, this possibly in part because a number of tools had been used not all of which were specific to asthma. Overall, two studies found no significant difference in disease specific quality of life scores, two found a significant improvement, and the fifth was equivocal.

Kim et al. reported quality of life outcomes in only two SLIT studies, both showed no improvement in quality of life [18].

### Lung function

Calamita et al. indicated that treatment by SLIT failed to show a significant improvement in FEV1%: SMD of 1.48 (95% CI 0.13–2.82) among 144 patient in four of the studies that investigated this outcome. FEF 25–75% also did not achieve statistical significance: SMD 1.06 (95% CI 0.40–1.72) among 42 patients in two studies [11]. Similarly, Tao et al. found, from five studies, no improvement in FEV1% pooled SMD of 0.49 (95% CI −0.36 to 1.34; P = 0.26) in the SLIT group [16].

### Environmental exposure chamber or bronchial allergen challenge

Calamata et al. reported that there was no significant improvement in bronchial provocation tests in the SLIT group, but no data were presented [11]. Normansell et al. found 11 studies which used the methacholine provocation test. Data from four of these trials were pooled; meta-analysis failed to show any evidence of benefit: SMD 0.69, 95% CI −0.04 to 1.43, with a high level of heterogeneity (I^2^ = 76%) [15].

### Safety

Calamita et al. reported adverse effects reported in 20 studies enrolling 1501 patients. Only mild adverse events were seen, the majority resolving without the need for treatment [11]. The relative risk of adverse effects was 1.83 (95% CI 1.40–2.40) and RD was 0.07 (95% CI 0.04–0.10) with a number needed to harm (NNH) for AIT of 14.3.

Normansell et al., from 22 RCTs, reported that serious adverse events were uncommon, occurring in 1 in 100 patients using SLIT (RD 0.001, 95% CI −0.008 to 0.010; moderate-quality evidence) [15]. When they looked at all AEs, however, they showed an increase of AEs in the SLIT group compared to the placebo with an OR of 1.70 (95% CI 1.21–2.38). Most of these AEs were however mild.

Tao et al., also concluded that the AEs were mild such as mouth and throat itchiness, redness and swelling [16]. Pooled data analysis through meta-analysis resulted in a significant risk in a RR 2.23 (95% CI 1.17–4.2; P = 0.01) with high level of heterogeneity (I^2^ = 75%). Kim et al. found in 12 SLIT studies a rate of local reactions from 0.2–50% in the treatment group but 6–25% in the placebo group [18]. Systemic reactions were common, but not life threatening and occurred in both treatment and placebo groups.

## Reviews including both SCIT and SLIT studies

Two systematic reviews examined the effect of SLIT versus SCIT on asthma. Kim et al. looked solely at children and included 27 trials: 12 SLIT and 12 SCIT, the results of which have been discussed above under the appropriate headings; there were a further three trials comparing the two treatment routes [18]. Chelladurai et al. looked at a head-to-head comparison of the two routes of administration [12].

### Asthma symptom score

Kim et al., using a narrative synthesis, found that in the studies that looked at SLIT versus SCIT there was no conclusive evidence to favor one route of administration over the other in terms of symptom scores [18].

A comparison between SCIT and SLIT was undertaken in the review by Chelladurai et al., which included four studies of asthma patients all using HDM immunotherapy [12]. They demonstrated a greater reduction in asthma symptoms from three studies with SCIT compared to SLIT, whereas one study showed greater reduction in symptoms with SLIT. All of these studies were judged to be of moderate quality.

### Asthma medication score

Kim et al. presented results from three studies of HDM immunotherapy directly comparing SLIT with SCIT, but there was no conclusive evidence to favor one route of administration over the other in terms of reducing medication scores. Two studies were described as favoring SCIT over SLIT for improving medication use, whereas one study favored SLIT.

Chelladurai et al., found that when comparing the HDM studies two studies favored SCIT in reducing medications usage while two favored SLIT [12].

### Asthma control

No results were available for this outcome.

### Disease specific quality of life

No results were available for these outcomes.

### Lung function and environmental exposure chamber or bronchial allergen challenge

No results were available for these outcomes.

### Safety

In Kim et al. among the three studies (including 135 children) that examined SCIT versus SLIT, local reactions were reported in three patients receiving SLIT and in three patients receiving SCIT. No systemic reactions were reported in patients receiving SLIT. Among the patients that received SCIT, four experienced systemic reactions, including one anaphylaxis event (anaphylaxis was defined as flushing, wheezing and dyspnea requiring adrenaline) [18].

In the comparison between SCIT and SLIT, Chelladurai et al. indicated that eight studies reported on AEs, however due to the heterogeneity data could not be pooled [12]. Local reactions occurred both in SCIT and SLIT with no fatalities; however SLIT was associated with an increased frequency of local reactions (7–56%) compared with SCIT (20%). The only episode of anaphylaxis was reported in one study in a child treated with SCIT.

### Health economic outcomes

No results were available for this outcome.

## Discussion

### Statement of principal findings

We found clear evidence that AIT administered by the SCIT route is effective in improving medication and symptom scores. The evidence in relation to the effectiveness of SLIT for these outcomes was more mixed. It is interesting to note however, that the review by Compalati et al. [12] which looked only at HDM AIT shows significant reductions in both symptom and medication scores for asthma compared to the review by Calamita et al. [11] which looks at a number of allergens which shows no significant reduction when considering the same outcomes. In terms of lung function no positive result could be concluded for either SCIT or SLIT. With regards to BHR, some of the studies showed an improvement in the SCIT group, but no clear conclusions could be drawn and no improvement in the SLIT group could be demonstrated for this outcome. There was considerable variation in results dependent upon which allergen was used and whether multiple or single allergens were administered with single allergen AIT faring more favorably. The two systematic reviews by Kim et al. [18] and Chelladurai et al. [12] which compared the two routes of administration could not conclusively show any difference between the effectiveness of SLIT and SCIT. Furthermore, it was difficult to compare results from these two reviews due to the heterogeneity between them including the fact that one was focused on the pediatric population only whilst the other looked at both adults and children. Furthermore, although they both looked at AIT for HDM, they also looked at different allergens with one concentrating on tree mix and the other pollens. Across all of the reviews, there was considerable variation in results dependent upon which allergen was used and whether multiple or single allergens were administered.

Safety is a prime concern with any treatment and the safety profile of SLIT compares more favorably to SCIT particularly in relation to the risk of systemic reactions. However, no fatalities were reported with either route of administration.

There were very few studies which considered and reported on disease specific quality of life as a study outcome. As a result due to the paucity of data present no conclusions can be drawn. This is therefore clearly an area that warrants further enquiry. Studies that considered this outcome used many different tools to assess quality of life some of which were not disease specific. This is another area where uniformity of reporting is urgently required.

### Strengths and limitations

We believe this is the first such synthesis of data from systematic reviews on AIT that has been undertaken. We used standard systematic overview techniques, which will have helped to minimize the risk of bias.

There are nonetheless some limitations of systematic overviews which should be considered when interpreting the results. First is in relation to the quality of studies that were included in the individual systematic reviews, many of which were at moderate or high risk of bias. Second is the wide variety of studies included within these systematic reviews, the majority of which are dated. They included patients with varying severities of asthma, allergies and treatment with single or multiple allergens and with treatment regimens of varying length and follow-up. Furthermore, there was no standardization of outcomes measured and even when outcomes overlapped there was still no standardization of measurements taken. This heterogeneity of studies may in part account for the varied results that were seen. Finally, meta-analysis was mainly confined in the studies that investigated SLIT rather than SCIT.

## Conclusions

This systematic overview has identified a substantial evidence base investigating the effectiveness and safety of AIT for allergic asthma, this showing that overall this treatment modality has the potential to improve medication and symptom scores. There was some indirect evidence to suggest that the effectiveness of SCIT may be superior to SLIT, but that the safety profile of SLIT is superior in relation to systemic AEs. We found no evidence in relation to cost-effectiveness considerations and equivocal and little or no evidence in relation to many of our secondary effectiveness outcomes of interest. A follow-on, more up-to-date evidence synthesis of primary studies may help to provide further clarity on the effectiveness, safety and cost-effectiveness of AIT.

## Appendix

Search strategy 1

(MEDLINE, EMBASE)

1. exp asthma/
2. asthma.mp.
3. asthmatic children.mp.
4. acute asthmatic attack.mp.
5. asthma control.mp.
6. asthma exacerbations.mp.
7. wheez*.mp.
8. respiratory hypersensitivity/
9. bronchial disorder.mp.
10. hyper-responsiveness wheez*.mp.
11. lung function.mp.
12. ventilatory function.mp.
13. FEV.mp.
14. FEF.mp.
15. FVC.mp.
16. PEF.mp.
17. bronchial hyperreactivity.mp.
18. airway hyperreactivity.mp.
19. bronchial responsiveness.mp.
20. airway responsiveness.mp.
21. or/1-20
22. exp Desensitization, Immunologic/
23. exp Immunotherapy/
24. desensiti?ation.mp.
25. (immunotherapy or allergen immunotherapy or oral immunotherapy).mp.
26. subcutaneous immunotherapy.mp.
27. sublingual immunotherapy.mp.
28. specific immunotherapy.mp.
29. Or/22-28
30. exp Intervention Studies/
31. intervention studies.mp.
32. exp Clinical Trial/
33. (trial or clinical trial).mp.
34. Exp Randomized Controlled Trial/
35. randomi?ed controlled trial.mp.
36. exp Placebos/
37. placebos.mp.
38. exp Random allocation/
39. random allocation.mp.
40. random*.mp.
41. exp Double-blind method/
42. double-blind method.mp.
43. double-blind design.mp.
44. exp Single-blind method/
45. single-blind method.mp.
46. single-blind design.mp.
47. triple-blind method.mp.
48. search:.tw.
49. review.pt.
50. systematic review.tw.
51. meta analysis.mp,pt.
52. case series.mp.
53. (case$ and series).tw.
54. cost:.mp.
55. cost effective:.mp.
56. cost utility:.mp.
57. exp Health Care Costs/
58. (costs and costs analysis).mp.
59. economic evaluation*.mp.
60. ((cost effective* adj1 analys*) or cost minimi?ation analys* or cost benefit analys* or cost utility analys* or cost consequence analys* or finances).mp.
61. Or/30-60
62. 21 and 29 and 61

Search strategy 2

(Cochrane library, HTA, EED, CINAHL, ISI Web of Science, TRIP)

(Asthma or acute asthmatic attack or wheez* or respiratory hypersensitivity or bronchial disorder or hyper-responsiveness wheez* or lung function or ventilatory function or bronchial hyperreactivity or airway hyperreactivity or bronchial responsiveness or airway responsiveness)

AND

(Immunologic, desensiti* or immunotherapy or oral immunotherapy or allergen immunotherapy or specific immunotherapy or subcutaneous immunotherapy or sublingual immunotherapy)

AND

(Intervention stud* or experimental stud* or trial or clinical trial* or randomi* controlled trial or random allocation or single blind method or double blind method or triple blind method or random* or systematic review or meta-analysis or case series or economic evaluation* or cost effective* analys* or cost minimi?ation analys* or cost benefit analys* or cost utility analys* or cost consequence analys* or finances)
